# Resuscitation of the Newborn: Simulating for Confidence

**DOI:** 10.7759/cureus.790

**Published:** 2016-09-20

**Authors:** Phil J Peacock, Anna Woodman, Wendy McCay, Sarah E Bates

**Affiliations:** 1 Department of Paediatrics, Great Western Hospital, Swindon; 2 The Academy, Great Western Hospital, Swindon

**Keywords:** neonatology, resuscitation, medical simulation, medical education

## Abstract

**Introduction:**

Non-pediatric trainees working in pediatrics in the UK are expected to attend newborn deliveries and provide initial newborn life support if needed. In Swindon, new junior doctors receive a 90-minute teaching session at the start of their pediatrics rotation, but the content has not previously been standardized, and it may be several weeks before a doctor attends a newborn delivery. Thus, the confidence and competence in newborn resuscitation of doctors attending deliveries can vastly vary.

**Methods:**

A standardized teaching package was developed as part of the pediatrics induction program. This includes an interactive lecture on the physiology of the newborn, skills stations, and mini-simulations to consolidate skills. This is accompanied by a program of regular neonatal mini-simulations as part of the departmental morning teaching program. These sessions allow junior doctors to practice their skills in a safe, simulated environment and reinforce the newborn life support pathway.

**Results:**

Qualitative and quantitative feedback was sought following delivery of the induction training session. Junior doctors were asked to rate their confidence before and after the induction session using Likert scales from 1 (least confident) to 5 (most confident). Median confidence in attending term deliveries increased from 2 (range 1 - 4) to 4 (2 - 5), P=0.008. There was evidence that confidence was maintained at one month following induction.

**Conclusions:**

A simulation program has been successful at improving confidence among junior doctors in attending newborn deliveries. This has the potential to improve patient care and trainees’ experiences of their pediatrics placement.

## Introduction

In the UK, junior doctors rotate through pediatric clinical posts as part of the generic foundation program and general practice (GP) specialty training. These doctors are usually expected to take on the same role as and work alongside pediatric specialist trainees in their first three years of training. Non-pediatric trainees are often required to hold the ‘delivery bleep’ which involves attending medium-to-high risk deliveries and starting emergency resuscitation if needed. Support is available from senior pediatric trainees and consultants, but this may not always be immediately available, especially outside of the normal working day where consultants are non-resident and the senior trainee may be covering multiple clinical areas.

The Great Western Hospital, Swindon, is a district general hospital in the UK, with an annual birth rate of approximately 4500. The junior pediatric team currently is comprised of two foundation year-2 (FY2) trainees, eleven GP trainees, and two first-year pediatric trainees. In the past, many of the GP and FY2 trainees rotating through the pediatrics department have reported anxiety around attending newborn deliveries, and the expectation of initiating newborn life support if needed. Resuscitation of the newborn has been covered as part of the local departmental induction, but the session content has previously been variable, and as exposure to clinical situations varies between trainees, there may be several months before the skills learned are needed in real life.

Simulation is increasingly being used in pediatrics and neonatology as a way to improve knowledge and skills, and prepare individuals and teams for emergency situations [1-3]. Challenges to this, however, include staff engagement with training, finding time within the busy working day (both faculty and staff), and availability of equipment [4].

## Materials and methods

A standardized induction program was developed to help ensure all new trainees are provided the initial knowledge and skills required for competence in newborn resuscitation. The first induction session is 90 minutes in length, with the induction for subsequent groups increased to 120 minutes on the basis of participant feedback. The program includes a brief, interactive lecture on newborn physiology, skills stations using low-fidelity manikins (airway management, delivering inflation/ventilation breaths, chest compressions), and mini-simulations to consolidate learning.

To help trainees maintain their skills and confidence throughout their placement, a program of regular mini-simulations were developed which are run one or two times per month. Scenarios include basic airway management, meconium at birth, and a baby born with an unexpectedly low Apgar score requiring full resuscitation. Initial scenarios were piloted and adapted on the basis of feedback prior to the formal launch of the program. Ongoing mini-simulation sessions are approximately 20 minutes in length (2-minute pre-brief, 5-10-minute scenario, 10-minute debrief) and have been incorporated into the pre-existing departmental teaching program. This has enabled trainees to attend without an additional pressure on their time. The importance of confidentiality and a safe learning space are emphasized to encourage participant involvement.

To measure the quality and impact of the training program, trainees were asked to complete feedback forms prior to induction, following induction, and one month into their placement. Verbal consent was obtained from all participants. Doctors were asked to rate their overall confidence in attending newborn deliveries (primary outcome) as well as in particular aspects of newborn resuscitation (secondary outcomes) using Likert scales from 1 (least confident) to 5 (most confident). There was also the opportunity to provide free-text comments. Participants were asked to include a three-digit identifier known only to themselves to enable matching of pre- and post-induction scoring while maintaining confidentiality. Pre- and post-induction confidence scores were compared using a Wilcoxon signed-rank test, with the threshold for statistical significance set at 0.05 for the primary outcome. For the five secondary outcomes, a Bonferroni correction was applied to account for multiple comparisons [5], giving a significance threshold of 0.01.

## Results

The formal program was launched in February 2016, with six junior doctors attending the structured induction program. A further three doctors were inducted in April 2016. All doctors completed pre- and post-induction questionnaires; five doctors (55%) completed follow-up questionnaires at one month.

Of the nine participating doctors, two were FY2s, and seven were GP trainees. One doctor had previously completed the European Pediatric Life Support course, one had completed the Pediatric Immediate Life Support course, and two had previously received training in Pediatric Basic Life Support. No doctors had previously attended a Newborn Life Support course or any other newborn-specific training. Three doctors (33%) had previously worked in pediatrics.

Median confidence in attending newborn deliveries significantly increased following the induction program and this confidence level was maintained at one month (Table 1). Similar results were seen for the secondary outcomes, with a statistically significant increase in confidence in 4 of the 5 specific skill areas. 

**Table 1:** Confidence Scores in Aspects of Newborn Resuscitation Confidence scores are median (range)

	Pre-induction (n=9)	Post-induction (n=9)	One Month (n=5)	P Value (Pre vs Post)
Attending newborn deliveries	2 (1-4)	4 (2-5)	4 (4-5)	0.008
Knowing newborn life support algorithm	2 (1-4)	4 (4-5)	4 (4-5)	0.009
Delivering newborn life support	2 (1-3)	4 (3-4)	4 (4-5)	0.007
Setting up and using resuscitaire	1 (1-3)	4 (3-5)	5 (4-5)	0.007
Basic airway maneuvers	2 (1-3)	4 (4-4)	5 (4-5)	0.007
Cardiac compressions	3 (1-4)	4 (3-4)	4 (4-5)	0.08

Qualitative comments following the induction program included "[it] was good to have time to practice individually and ensure we had mastered the appropriate skills" and "[I] feel much more confident but until I've actually done it I think I will still carry a degree of fear."

## Discussion

A structured induction program, including manikin-based skills learning and short simulated scenarios, increased confidence in attending newborn deliveries among junior doctors rotating through pediatrics. Embedding regular simulation sessions within a departmental teaching program has been well-received, and there is some evidence that doctors are maintaining their confidence in attending deliveries and performing key techniques. One free-text comment highlighted some remaining anxiety following the induction session; it is hoped the regular simulation sessions help to reinforce the learning and increase confidence, reducing any on-going fear.

Less than half of the doctors had attended formal pediatric resuscitation training prior to starting work, and none had received newborn-specific training. The cost of Newborn Life Support courses and the time out-of-work required to attend means it is not possible for many district general hospitals to send new staff on these formal courses. Thus training programs such as this are needed to ensure clinicians have the knowledge and skills necessary to manage emergency situations. The demonstrated increase in confidence and skills in attending deliveries and initiating newborn resuscitation has clear benefits in terms of patient care and will also improve the experience of junior doctors in the department.

A strength of the induction program is that the small-group (maximum three per group) skills stations require trainees to demonstrate skills to an experienced faculty member prior to completing the session. Additionally, the regular simulation sessions are run by experienced pediatric clinicians who are able to assess techniques and provide formative feedback to junior doctors.

As this is a new project, the number of participants so far has been relatively low, but the results from those who have taken part are encouraging and support the ongoing delivery and development of the simulation program. The results are potentially limited by the use of self-reported measures of confidence; it is possible that participants may over-report their confidence or that confidence may not correlate with clinical competence. However, the small-group teaching and expert feedback from skills practice and simulation scenarios help ensure that trainees are aware of expectations and, therefore, able to assess their confidence and skills appropriately, reducing the potential impact of unconscious incompetence on the results. Anonymous identifiers, where faculty are unable to identify an individual's responses, were used to reduce possible self-report bias.

Completion of one-month follow-up questionnaires was 55%, which limited the interpretation of these results. Follow-up questionnaires were sent by email in an effort to improve completion, as shift patterns can make it difficult to contact individuals face-to-face. However, in future work, a dual approach will be considered to try and maximize completion rates.

This program has focused on the training needs of junior doctors who are relatively inexperienced in pediatrics. However, there is also a need for other health professionals working in maternity areas to be confident in managing newborn emergencies, and good team working across professions is crucial for high-quality clinical care [6]. It is hoped to expand the current simulation program to include nursing and midwifery staff with an added focus on human factors and team working.

## Conclusions

Our program of neonatal simulation training has increased confidence among junior doctors in attending deliveries and managing newborn emergencies. This simulation program will continue to be developed, taking into account feedback from those participating. Areas for future development include the use of an objective skills measure to provide feedback for trainees and better measure the impact of the training program. We will also seek to collaborate with other hospitals within our training region to share our good practice and look at ways to standardize training between hospitals. Other simulation programs within the department already have multi-professional involvement, and we are keen to expand this program to neonatal nursing and midwifery staff.
